# The complete mitochondrial genome of the hybrid grouper (*Cromileptes altivelis♀* × *Epinephelus lanceolatus♂*) with phylogenetic consideration

**DOI:** 10.1080/23802359.2017.1303346

**Published:** 2017-04-05

**Authors:** Jiaxing Chen, Zhifeng Ye, Zeshu Yu, Jing wang, Peisi Li, Xiao Chen, Yun Liu, Yong Zhang

**Affiliations:** State Key Laboratory of Biocontrol, Institute of Aquatic Economic Animals, and the Guangdong Province Key Laboratory for Aquatic Economic Animals, Sun Yat-Sen University, Guangzhou, P.R. China

**Keywords:** Hybrid grouper, mitochondrial genome, phylogenetic analysis

## Abstract

The complete mitochondrial genome of the Hybrid grouper (*Cromileptes altivelis♀ × Epinephelus lanceolatus♂*) was presented in this study. The mitochondrial genome is 16,501 bp long and consists of 13 protein-coding genes, 2 rRNA genes, 22 tRNA genes, and a control region. The gene order and composition of Hybrid grouper mitochondrial genome was similar to that of most other vertebrates. The nucleotide compositions of the light strand are 26.24% of A, 15.68% of C, 29.07% of T, and29.01% of G. With the exception of the NADH dehydrogenase subunit 6 (*ND6*) and eight tRNA genes, all other mitochondrial genes are encoded on the heavy strand. The phylogenetic analysis by maximum-likelihood (ML) method shows that the hybrid grouper has closer relationship to *C. altivelis*.

*Cromileptes altivelis* and *Epinephelus lanceolatus* both belong to Serranidae in the order Perciformes. They are both important commercial mariculture fish species, due to their excellent food quality, abundant nutrients, and high market value. The hybrid grouper (*C. altivelis*♀ × *E. lanceolatus*♂) exhibits significant growth superiority over its female parent, which made it a promising farmed species in grouper aquaculture industry in China. It presents a good combination of beneficial traits from both parent species, and had higher growth rates compared to other grouper species. But the molecular mechanisms of its heterosis still remain poorly understood. Therefore, the purpose of this study was to sequence the complete mitochondrial genome of the hybrid grouper (C. altivelis♀ × E. lanceolatus♂) by using the next-generation sequencing (NGS) techniques strategy (Xie et al. 2015), in order to develop new DNA markers for the studies on population genetics of the hybrid grouper. The specimen was obtained from Daya Bay Fishery Development Center, Guangdong, China. The total genomic DNA was extracted from the fin of the fresh fish using the salting-out procedure (Howe et al. 1997).

The entire sequence of Hybrid grouper (*C. altivelis*♀ *× E. lanceolatus*♂) mitochondrial genome (Genbank accession number KY249560) is 16,501 bp in length, consisting of 13 protein-coding genes, 2 ribosomal RNAgenes (12S rRNA and 16S rRNA), 22 transfer RNA genes (tRNA), and 1 control region (Figure 1), which was the same as the typical vertebrates (Wang et al. 2008). Most of the genes were encoded on the heavy strand, with only the NADH dehydrogenase subunit6 (*ND6*) and eight tRNA genes (*Gln*, *Ala*, *Asn*, *Cys*, *Try*, *Glu*, *Pro*, *Ser* (TGA)) encoded on the light strand. Overall nucleotide compositions of the light strand are 26.24% of A, 15.68% of C, 29.07% of T, and 29.01% of G. However, the most representative base is A and the bias against G was observed, similar to the base compositions of mitochondrial genome of other teleosts.

**Figure 1.**
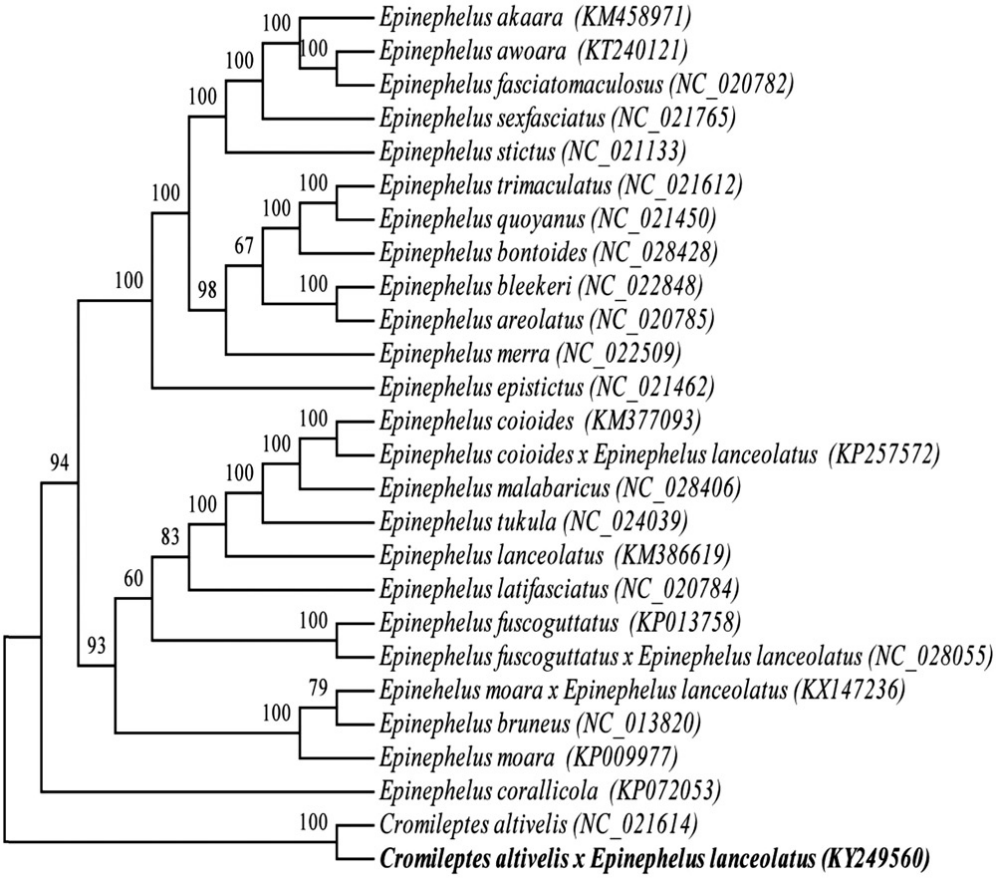
The ML phylogenetic tree of Perciformes species. Numbers on each node are bootstrap values of 100 replicates.

All the protein-coding genes began with an ATG start codon except for *COX1* started with GTG, and *ATP6* started with TTG. Three types of stop codons revealed were TAG (*ND5*, *ND6*), TAA (*ND1*, *COX1*, *ATP8*, *ATP6*, *ND4L*), TA– (*COX3*), and T–– (*ND2*, *COX2*, *ND3*, *ND4*, *CYTB*). The 12S and 16S rRNA genes were located between the *tRNA-Phe* (GAA) and *tRNA-Leu* (TAA) genes, and are separated by the *tRNA-Val* gene with the same situation found in other vertebrates. Most genes are either abutted or overlapped. The 22 tRNA genes vary from 67 to 75 bp in length. All these could be folded into the typical cloverleaf secondary structure although numerous non-complementary and T–G base pairs exist in the stem regions. There are 10 intergenic spacers (total 74 bp) varying from 1 to 39 bp in length and 5 gene overlaps (total 23 bp), the largest of which is 10 bp between *ATP8* and *ATP6*.The control region was 805 bp in length, located between *tRNA-Pro* (TGG), and *tRNA-Phe* (GAA) gene. The nucleotide composition of control region was 33.79% of A, 18.01% of C, 14.78% of G, and 33.42% of T.

The phylogenetic position of the hybrid grouper *C. altivelis*♀ *× E. lanceolatus*♂ was reconstructed with the complete mtDNA sequences from 26 species of Perciformes by using the maximum-likelihood (ML) methods (Kumar et al. 2004). As shown in Figure 1, the hybrid grouper *C. altivelis*♀ *× E. lanceolatus*♂ has closer relationship to *C. altivelis*.
